# A Fast and Accurate Lane Detection Method Based on Row Anchor and Transformer Structure

**DOI:** 10.3390/s24072116

**Published:** 2024-03-26

**Authors:** Yuxuan Chai, Shixian Wang, Zhijia Zhang

**Affiliations:** 1 School of Artificial Intelligence, Shenyang University of Technology, Shenyang 110870, China; yuxuanchai@smail.sut.edu.cn (Y.C.); zhangzj@sut.edu.cn (Z.Z.); 2 Shenyang Key Laboratory of Information Perception and Edge Computing, Shenyang 110870, China

**Keywords:** lane detection, row-anchor-based method, transformer, structural loss, expectation loss

## Abstract

Lane detection plays a pivotal role in the successful implementation of Advanced Driver Assistance Systems (ADASs), which are essential for detecting the road’s lane markings and determining the vehicle’s position, thereby influencing subsequent decision making. However, current deep learning-based lane detection methods encounter challenges. Firstly, the on-board hardware limitations necessitate an exceptionally fast prediction speed for the lane detection method. Secondly, improvements are required for effective lane detection in complex scenarios. This paper addresses these issues by enhancing the row-anchor-based lane detection method. The Transformer encoder–decoder structure is leveraged as the row classification enhances the model’s capability to extract global features and detect lane lines in intricate environments. The Feature-aligned Pyramid Network (FaPN) structure serves as an auxiliary branch, complemented by a novel structural loss with expectation loss, further refining the method’s accuracy. The experimental results demonstrate our method’s commendable accuracy and real-time performance, achieving a rapid prediction speed of 129 FPS (the single prediction time of the model on RTX3080 is 15.72 ms) and a 96.16% accuracy on the Tusimple dataset—a 3.32% improvement compared to the baseline method.

## 1. Introduction

The advent of AlexNet [1] marked a significant milestone in the efficacy of deep learning, demonstrating notable accomplishments in lane detection and diverse application domains. Despite these advancements, challenges persist in deep learning-based lane detection, particularly in scenarios characterized by low light conditions, intense illumination, and disrupted or obstructed lane markings, necessitating an augmented capacity for robust global feature extraction. Furthermore, the exigencies of real-time performance in lane detection pose stringent requirements, urging the enhancement of the model’s inference speed while ensuring precision. This imperative optimization aims to conserve computational resources, fostering efficiency in other facets of Advanced Driver Assistance Systems (ADASs).

In addressing the challenge of indistinct lane lines, prevalent methodologies involve carrying out a holistic regression of lane lines through the extraction of global features from input images, coupled with the design of network structures and loss functions incorporating prior knowledge of the slender and continuous nature of lane lines. Enet-SAD [2] achieves comprehensive lane line regression by facilitating the retention of contextual information across deeper network layers, allowing information to flow seamlessly between these layers. CLRNet [3] adopts the Feature Pyramid Network (FPN) [4] structure, employing the ROIGather module to preemptively extract lane line features, thereby enhancing the model’s capacity to capture global features—a distinction that positions it as the most effective model among contemporary methods. Conversely, CondLaneNet [5] utilizes the FPN structure alongside a self-attention mechanism for global feature extraction; however, the exclusive reliance on the self-attention mechanism, without a comprehensive Transformer encoder–decoder structure, diminishes its global feature extraction capability. Moreover, a lot of parameters in the self-attention mechanism contribute to a partial slowdown in network’s prediction speed. SCNN [6] and RESA [7] leverage a priori knowledge of lane line morphology and employ a message-passing mechanism to gather global features; however, their requirement for pixel-level predictions substantially impedes the network’s prediction speed. In contrast, the Ultra Fast network incorporates a structural loss function based on the robust a priori understanding that lane lines exhibit continuity and typically feature modest slopes.

When addressing real-time constraints in model performance, conventional lane detection approaches rooted in segmentation methods prioritize accuracy. For instance, Lu et al. introduced a robust semantic segmentation network [8] that effectively segments key frames, coupled with a streamlined optical flow estimation network for tracking non-key frames. Despite achieving commendable accuracy and robustness, the real-time performance of semantic-based segmentation methods is compromised as they necessitate the classification of each pixel point. In recent years, row-anchor-based approaches in lane detection have emerged as favored solutions for real-time applications. Exemplary models, such as E2E-LMD [9], Ultra Fast [10], Ultra Fast V2 [11], and CondLaneNet [5], have demonstrated noteworthy real-time performance. The distinctive characteristic of row-anchor-based methods involves an initial grid division of input images, followed by the classification of individual grids within each row, thereby obviating the need to classify every pixel point. This strategic approach ensures robust real-time performance while maintaining accuracy.

Grounded in the success of models such as VIT [12], DETR [13], and other Transformer-based architectures in the realm of computer vision, the incorporation of Transformer structures has yielded notable advancements. Lane Transformers [14] have specifically integrated the Transformer encoder–decoder structure into lane line detection tasks, showcasing commendable real-time performance. This study introduces a novel network architecture by amalgamating the row-anchor-based method with the Transformer encoder–decoder structure, leveraging global information comprehensively for enhanced lane line detection in intricate scenarios while upholding the efficient real-time performance that is inherent to row-anchor-based lane detection methods. The proposed approach employs ResNet [15] as the backbone to extract input image features. In instances of unclear or imperceptible lane lines, inspired by GANet [16], a Transformer encoder–decoder structure is employed to extract global features and discern the set of lane line coordinates based on the row-anchor-based lane detection methodology. Diverging from conventional methods [17], pooling is adopted as the token module within the transformer structure, thereby reducing model parameters and augmenting the prediction speed.

Drawing inspiration from the architectural design of the Ultra Fast network, our approach incorporates an auxiliary branch that is operative solely during training, enhanced through a Feature-aligned Pyramid Network (FaPN) [18]. An FaPN strategically utilizes the middle and output layers of the backbone network to extract additional global features, thereby enhancing the network’s prediction accuracy without incurring additional prediction time overhead. Building upon the structural foundation of Ultra Fast’s loss function, we refine the structural loss to more effectively harness the robust a priori understanding that lane lines exhibit a slender and continuous nature. Additionally, we introduce a novel expectation loss to further elevate the model’s accuracy. The efficacy of our proposed model is demonstrated on the Tusimple dataset, wherein it achieves a notable accuracy of 96.16%. The experimental results underscore the model’s adeptness in achieving both high accuracy and meeting real-time requirements, notably achieving a prediction speed of 129 frames per second (FPS). Our primary contributions are summarized as follows:

This study introduces an innovative framework for row-anchor-based lane detection by employing a Transformer encoder–decoder structure. This framework addresses challenges associated with lane detection in scenarios characterized by indistinct or absent lane lines, all while preserving real-time performance and optimizing the utilization of contextual information. The proposed method has achieved certain progress in the field of lane detection, demonstrating a balance between accuracy and real-time performance.

This study formulates a novel loss function by enhancing the structural loss derived from the Ultra Fast network. Additionally, we incorporate an expectation loss into the loss function framework. This nuanced approach contributes to the refinement and optimization of the loss function, demonstrating a methodological advancement in model training for improved performance and accuracy.

Comprehensive experimentation is undertaken to validate the efficacy of the proposed methodology. Our method approaches the current state of the art, achieving an accuracy of 96.16% on the Tusimple dataset. Furthermore, the fastest prediction speed achieved by our method reaches 129 FPS per second (the single prediction time of the model on RTX3080 is 15.72 ms). This confluence of high accuracy and a rapid prediction speed underscores the meticulous balance achieved by our methodology between computational efficiency and predictive precision.

## 2. Related Work

This section centers on the methodologies and accomplishments delineated by preceding researchers within the domain of lane detection by employing deep learning paradigms. The categorization of lane detection methods in this study is structured into five distinct categories, predicated on the manner in which previous authors have represented lanes. These categories encompass the following: 1. instance segmentation-based methods; 2. row-anchor-based methods; 3. line-anchor-based methods; 4. curve-based methods; and 5. key-point-based methods. This systematic classification serves as a foundational framework for the examination and synthesis of advancements in the field, facilitating a comprehensive understanding of the diverse approaches employed in deep learning-based lane detection.

### 2.1. Instance Segmentation-Based Methods

The instance segmentation-based method, a classical approach to lane detection, entails the classification of each pixel point within the input image to ascertain its association with a lane line. In contrast to conventional semantic segmentation methods that solely partition pixel points to identify the target for detection, the instance segmentation approach necessitates the categorization of pixel points encompassed by each lane line as distinct instances, thereby enabling the differentiation of multiple lane lines. However, due to the importance of classifying each pixel point, the prediction speed of lane detection when utilizing the instance segmentation-based method exhibits a notable disparity when compared to alternative methodologies. This disparity poses challenges in meeting the stringent real-time requirements that are essential for contemporary autonomous driving applications.

In 2018, Neven et al. introduced LaneNet [19], a seminal model in the domain of lane detection. Their pioneering work innovatively reframed the lane detection challenge into an instance partitioning problem, adopting an end-to-end training approach. Notably, they employed a clustering algorithm to dynamically determine the number of lane lines to be detected, eliminating the need for the artificial presetting of this parameter. In parallel, Pan et al. presented SCNN [6] in the same year, introducing Spatial CNN. This model leverages the inherent a priori knowledge of lane line shapes, which is particularly beneficial in addressing challenges posed by complex scenes featuring disrupted or obscured lane lines. These contributions mark significant strides in advancing the capabilities of lane detection models.

### 2.2. Row Anchor-Based Methods

In 2020, Qin et al. introduced the groundbreaking Ultra Fast Lane Detection model [10], a pioneering model in the realm of lane detection based on row anchor methods. Notably, this approach excels in achieving rapid prediction speeds compared to alternative lane detection methodologies, thereby meeting both accuracy and real-time requirements in practical scenarios. The core methodology of the row anchor-based method involves the grid-based partitioning of the input image. This entails determining the presence of lane lines in each row and subsequently evaluating individual cells within each row to ascertain the specific location of lane lines.

Ultra Fast Lane Detection innovatively introduced a streamlined approach to line classification, markedly reducing the computational costs and achieving an unparalleled prediction speed of 320 frames per second (FPS). The network architecture incorporates an auxiliary branch, grounded in instance segmentation, and it is exclusively operational during training to enhance the prediction accuracy without compromising speed. In 2022, Qin et al. proposed an enhanced version, Ultra Fast Lane DetectionV2, which eschews the auxiliary branches of the original model and adopts a hybrid anchor. This hybrid anchor dynamically selects between row and column anchors based on slope analysis, further enhancing detection accuracy [11]. In the same year, Liu et al. presented the CondLaneNet network [5], introducing a two-step instantiation process for lane lines. Initially, a suggestion header proposes the starting point of each lane line, followed by the addition of an offset map to row-anchor-based methods using a conditional shape header. This innovative approach significantly improves prediction accuracy while maintaining real-time performance. The accomplishments of CondLaneNet affirm the viability of row-anchor-based methods in the domain of lane detection.

### 2.3. Line-Anchor-Based Methods

Lane detection employing the line anchor method primarily capitalizes on a priori knowledge concerning lane lines by devising a line anchor akin to the anchor box concept in YOLO [10]. Subsequently, the model predicts the offset of the line anchor to derive accurate lane line predictions. Owing to the integration of a priori line anchors, these methodologies consistently demonstrate high accuracy. Notably, the DLA-34 iteration of CLRNet [3] stands out as the most accurate among contemporary models, attesting to the efficacy of the line anchor approach in enhancing precision in lane detection tasks.

In 2019, Li et al. introduced Line-CNN [20], marking the inception of the first lane detection method based on the line anchor concept inspired by Faster-RCNN [21]. Building on this line anchor approach, Tabelini et al. presented LaneATT [22] in 2021, incorporating a novel self-attentive mechanism. This mechanism intelligently fuses high and low-level features, addressing challenges posed by lane line occlusion in detection tasks. Subsequently, in 2022, Zeng et al. proposed CLRNet [3], which is currently recognized as the most accurate lane detection model, albeit with room for improvement in real-time performance. The model features an ROI collection module that concurrently gathers global information at each feature layer of the backbone network. The prediction results at each layer can then be utilized as input for subsequent layers. Furthermore, CLRNet introduces a novel loss function, Line IoU, which holistically considers lane lines, contributing to its accuracy-centric approach in lane detection tasks.

### 2.4. Curve-Based Methods

Lane detection methodologies based on curve fitting leverage polynomial functions to model lane lines, inherently mitigating issues associated with lane line occlusion and obviating the necessity for post-processing. Earlier methods, such as PolyLaneNet, encountered challenges in achieving a balance between real-time performance and accuracy. Subsequent advancements, exemplified by BézierLaneNet and its successor networks, have notably addressed these challenges. The incorporation of BézierLaneNet and its successors demonstrates improvements in both real-time responsiveness and accuracy within the realm of curve-fitting-based lane detection methods.

In 2020, Tabelini et al. introduced PolyLaneNet [23], pioneering the utilization of polynomial curves as an alternative to the direct representation of lane lines through key points. Subsequently, in 2022, Feng et al. proposed BézierLaneNet [24], innovatively employing easily computable, stable, and transformable Bézier curves to represent lane lines. Notably, the model introduced a feature fusion module based on deformable convolution, leveraging a priori knowledge of lane line symmetry to enhance the fitting of lane lines. In the same year, Jin et al. presented EigenlaneNet [25], introducing a novel concept of feature lanes for the purpose of fitting lane lines. Building on this progression, in 2023, Chen et al. proposed BSNet [26], achieving notable accuracy while introducing the novel approach of fitting lane lines using b-sample curves. Distinguishing itself from Bézier curves, the b spline curve overcomes challenges associated with curve oscillation in high-order Bézier curves, difficulties in splicing, and limitations in local modifications. These successive contributions mark advancements in the representation and fitting of lane lines within curve-fitting-based lane detection methodologies.

### 2.5. Key-Point-Based Methods

Lane detection based on the key point detection method reframes the lane detection challenge as a sequence of key point prediction tasks, affording a more adaptable representation of lane lines. In 2021, Qu et al. introduced FOLOLaneNet [27], emphasizing the modeling of local patterns to achieve global structural prediction through a bottom-up approach. However, the substantial parameterization in this model compromises its real-time performance. Subsequently, in 2022, Wang et al. presented GANet [16], adopting a two-branch structure incorporating confidence features and offset features to enhance local accuracy. A notable innovation of GANet lies in its introduction of a global perspective for key point prediction, yielding higher accuracy compared to local prediction methodologies. These contributions represent advancements in the key point detection approach, showcasing efforts to balance local accuracy with computational efficiency.

## 3. Materials and Methods

**Figure 1:**
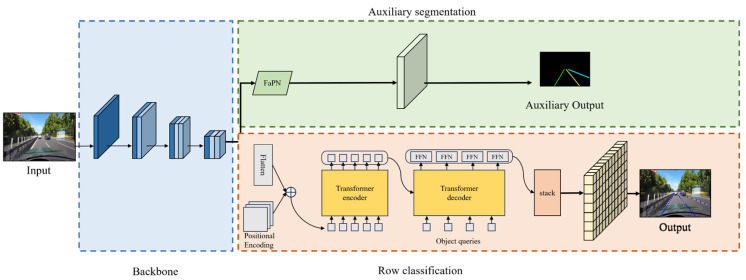
This figure illustrates the proposed network’s architecture. It starts with an input image being processed by a ResNet backbone for feature extraction. These features are then processed separately in two branches: row classification and auxiliary segmentation. In the row classification branch, the features are flattened and fed into a Transformer encoder with positional encoding. The Transformer decoder uses these encoded features along with learnable object queries to generate row anchor coordinate maps for four lane line instances. These maps are then combined to form the final lane representation map. Meanwhile, the auxiliary segmentation branch utilizes features from the backbone’s middle and output layers, employing a Feature-aligned Pyramid Network (FaPN) structure for segmentation-based lane detection. Notably, this auxiliary branch is only active during training.

The comprehensive network architecture, as delineated in Figure 1, can be dissected into three integral components. Firstly, the backbone employs ResNet as its foundational network, which is strategically employed for the extraction of input image features. Subsequently, the row classification branch incorporates a Transformer encoder–decoder structure, which is a pivotal element for predicting the lane representation map based on line classification methodologies. Lastly, the Auxiliary Segmentation Branch, which is exclusively operational during the training phase, utilizes the middle and output layers of the backbone network as input. By leveraging the instance segmentation method with a Feature Pyramid Attention Network (FaPN), this branch detects lane lines by aggregating contextual information. The inclusion of auxiliary branches holds paramount significance in the training process, enhancing the backbone network’s proficiency through the instance segmentation method. This augmentation results in improved prediction accuracy without incurring any compromise to the prediction speed. The elucidation of these network components showcases a meticulous design aimed at achieving optimal performance in lane detection tasks.

### 3.1. The Lane Representation

**Figure 2:**
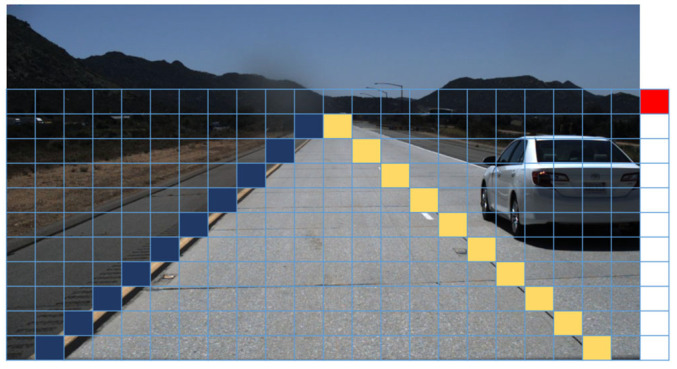
Lane representation map. The input image is pre-gridded, and for each lane instance, it is only necessary to determine which grid represents the lane in each row.

This study employs row-anchor-based methods for the representation of lane lines, as illustrated in Figure 2. These methods, showcased for their superior real-time lane detection capabilities in comparison to segmentation-based approaches, adopt a distinct paradigm. Given an input image with dimensions of length W and height H, the methodology involves creating a mesh by partitioning the image into a grid with w+1 rows and h columns. Subsequently, the lane detection problem is reformulated into a line classification challenge. Within this framework, each lane line instance is characterized by identifying the specific cells in each row that contain the lane line or marking the first cell if the row lacks a lane line. This transformation yields a comprehensive set of lane line coordinate points, exemplifying the efficacy of the row-anchor-based methodology in delineating lane lines in a manner conducive to real-time detection.

$$P_{i,j,:}=f^{ij}\left(X\right),\;\;\;\;\;i\in \left(1,C\right),h\in (1,h)$$

where $P_{i,j}$ is a $\left(w+1\right)$-dimensional vector representing the probability of (w+1) cells in the jth row of the ith lane line, C is the number of lane lines, and h is the number of row anchors.

### 3.2. Row Classification Branch

**Figure 3:**
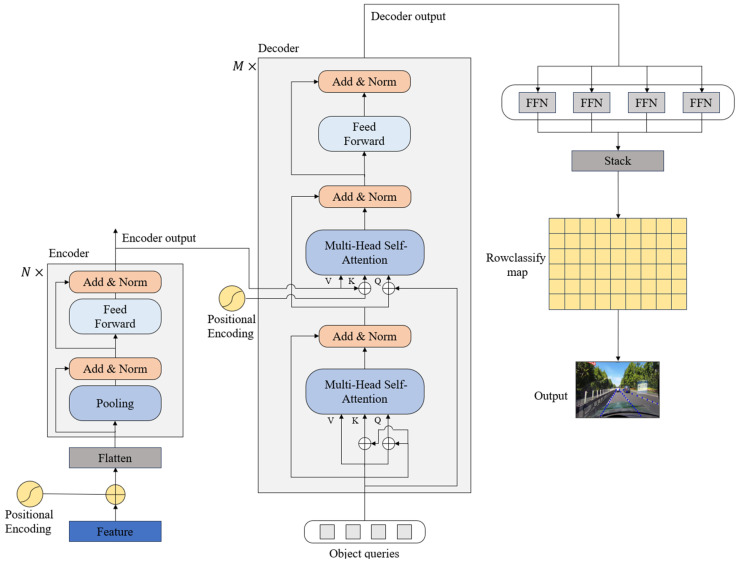
The schematic in Figure 3 outlines the row classification branch structure. Features from the backbone network, enhanced with positional embedding, enter the Transformer encoder. After passing through N encoders, the output is combined with object queries for further processing in the M-decoder Transformer module. The resulting feature maps then pass through four feed-forward networks, producing four coordinate maps for distinct lane line instances. These maps are concatenated to form the final lane representation map, revealing the branch’s intricate architecture within the larger network.

Lane lines, which are typically characterized by their elongated and slender configuration, often present challenges of indistinct or obscured visibility under real road conditions. To address this, an imperative is established for a lane detection methodology possessing robust contextual feature fusion capabilities. To fulfill this need, we employ a Transformer encoder–decoder structure as the architectural foundation for the lane detection head. This strategic choice facilitates the enhanced utilization of global features, thereby augmenting the model’s ability to discern and interpret the intricate details of lane lines within complex visual contexts. The structural instantiation of the row classification branch is illustrated in Figure 3, delineating the embodiment of this approach within the overall network’s architecture.

With the introduction of the Vision Transformer (VIT) [12] method, the Transformer architecture [28] has been incorporated into the realm of computer vision. Subsequently, the Detection Transformer (DETR) [13] model has demonstrated remarkable success in target detection tasks, showcasing the versatile capabilities of the Transformer architecture in extracting and fusing contextual features within the domain of computer vision. Despite these accomplishments, challenges persist in the form of a substantial number of parameters associated with the attention module. This abundance of parameters poses difficulties in adhering to the stringent real-time requirements that are essential for efficient lane detection. The existing body of literature, encompassing contributions such as those presented in references, attests to the ongoing challenges in reconciling the computational demands of attention modules with the real-time constraints inherent in the context of lane line detection.

The design of the encoder component draws inspiration from Mateformer. Contrary to conventional assumptions that attribute the performance of the transformer encoder primarily to its attention module, that analysis reveals that the overall architecture plays a more pivotal role. Considering the importance of real-time adherence in the lane detection task, we introduce a strategic modification: we employ pooling as the token module within the transformer encoder. This adaptation, denoted as the Poolformer encoder, is characterized by its capacity to curtail the number of model parameters. Simultaneously, it excels in extracting global features, thereby aligning with the demands of real-time processing in the context of lane detection. This innovative encoding approach contributes to a nuanced understanding of the transformer architecture’s efficacy beyond traditional attention-centric perspectives. The specific flow of the Poolformer encoder can be expressed as follows:

X=InputEmb(I)

$$Y=Pooling\left(Norm\left(X\right)\right)+X$$

$$Z=\sigma \left(Norm\left(Y\right)W_{1}\right)W_{2}+Y$$

where I denotes the input feature, X denotes the input of the PoolFormer encoder processed by InputEmbedding, Y denotes the output of the first block of the poolformer encoder, Z denotes the output of the PoolFormer encoder, σ is a non-linear activation function.

The schematic representation of the row classification branch structure is depicted in Figure 3. The intricate process unfolds as follows: Initially, features derived from the backbone network, augmented with positional embedding via flattening operation, serve as inputs to the Transformer encoder. Subsequently, these inputs traverse through the Transformer encoder module, comprising N Transformer encoders. The output, coupled with object queries, undergoes further processing within the Transformer decoder module, which consists of M Transformer decoders. The resultant feature maps from the Transformer decoder module traverse through four distinct feed-forward neural networks, yielding four individual coordinate maps. Each of these feature maps signifies the coordinate map corresponding to a specific lane line instance. The final step involves concatenating these four coordinate maps, representing different lane line instances, to generate the conclusive output—the lane representation map.

**Figure 4:**
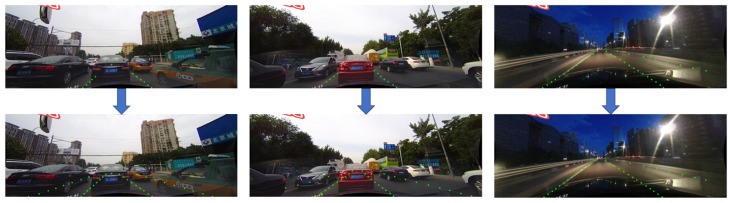
The upper part of Figure 4 shows the prediction results of directly inputting the backbone network output features into the FFN in the line classification branch, while the lower part demonstrates the prediction results obtained using the Transformer structure, as illustrated in Figure 3, in the line classification branch. The sample images are from the CULane public dataset and was taken in Beijing, China.

Our model, leveraging a transformer structure with superior global feature extraction capabilities in the line classification branch, excels in perceiving lane lines holistically. This enhanced perspective enables more precise predictions of lane line positions, especially in situations where lane lines are ambiguous, by effectively utilizing contextual features. Figure 4 demonstrates that, by incorporating the transformer structure, our model successfully predicts ambiguous lane lines with remarkable accuracy.

### 3.3. Auxiliary Segmentation Branches

**Figure 5:**
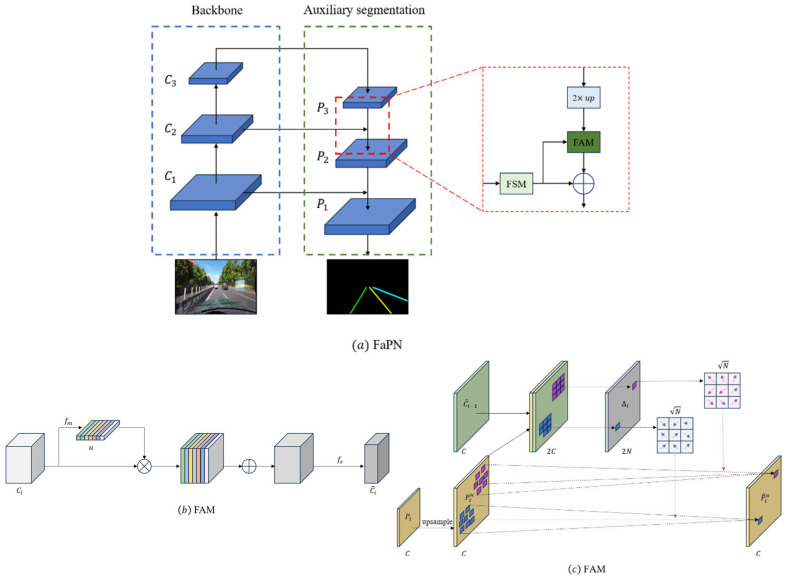
The auxiliary branch’s specific structure. The overall structure of the auxiliary branch is shown in (**a**). The auxiliary branch takes the backbone network intermediate and output layer features as the input, unlike traditional FPNs. The FaPN introduces the FAM module and the FSM module, and their structures are shown in (**b**) and (**c**), respectively.

In order to improve the robustness of the model to targets of different sizes, we use the features of the ResNet intermediate and the output layers of the backbone network as inputs, and we use the FaPN structure in the auxiliary branch to fuse the contextual information, which is shown in Figure 5.

The conventional Feature Pyramid Network (FPN) structure, designed to enhance model robustness across diverse target sizes by integrating deep semantic information with shallow detail information, encounters a challenge in terms of feature map alignment. The issue arises when the upsampled feature maps $P_{i}$ are combined with local feature maps $C_{i-1}$. This misalignment impedes the accurate integration of information, thus affecting the precision of the model. This consideration highlights a potential limitation in the conventional FPN structure with regard to the alignment of feature maps during the fusion process.

The Feature-aligned Pyramid Network (FaPN) structure demonstrates a capacity for adaptive learning across multiple scales, thereby incorporating heightened spatial details conducive to precise localization. Notably, the Feature Alignment Module (FAM) within the FaPN structure facilitates the learning of transform offsets for pixels, ensuring the alignment of feature maps at various layers. The architecture of the FAM module is delineated in Figure 5b. Additionally, a Feature Selection Module (FSM), represented in Figure 5c, is incorporated to accentuate shallow features so that they are replete with rich spatial details. Importantly, the auxiliary branch, a component that is integral to training procedures, remains inactive during prediction phases. This innovative approach, grounded in the re-parameterization concept, engenders an enhancement in detection accuracy without incurring any temporal overhead during prediction intervals. The FaPN structure thus emerges as a nuanced framework that is adept at optimizing feature maps for diverse scales and spatial intricacies, manifesting its utility in advanced object localization tasks.

### 3.4. Loss Function

The loss function can be divided into 4 parts, which are classification loss $L_{cls}$, expectation loss $L_{exp}$, shape loss $L_{shp}$, and auxiliary branch loss $L_{seg}$.

Classification loss $L_{cls}$: We consider the lane line detection problem as a row classification problem, where we only need to predict the probability that each block in each row contains lane lines, instead of classifying each pixel point, thus greatly reducing the prediction time.

$$L_{cls}=\sum_{i=1}^{C}\sum_{j=1}^{h}L_{CE}(P_{i,j,:},T_{i,j,:})$$

where $P_{i,j}$ represents the w+1 dimensional vector representing the lane position predicted by the jth line of the ith lane, $T_{i,j}$ represents the label value in the one-hot form of the jth line of the ith lane, $L_{CE}$ represents the cross-entropy loss, C represents the number of total lane instances, and h represents the number of line anchors.

**Figure 6:**
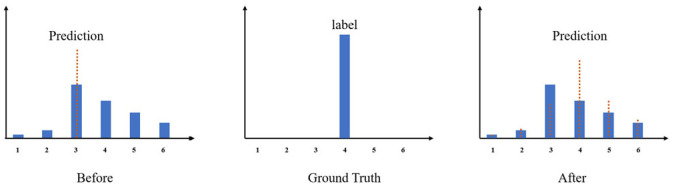
Expectation loss. The categorical loss takes only the category with the highest predicted probability as the prediction, while the predicted value of each category in the expected loss affects the prediction. The dotted line represents the confidence level corresponding to each horizontal coordinate.

Expectation loss $L_{exp}$: Inspired by UltraFasv2 we added an expectation loss in the loss function section to make the final prediction closer to the true value. Specifically, we do not want the final prediction result to depend only on the position of the cell with the highest probability in the prediction vector $P_{i,j}$, but rather, we want to make the predicted probability in each cell of the prediction vector $P_{i,j}$ have some influence on the final prediction value, as shown in Figure 6.

The expectation formula is as follows:

$$L_{exp}=\sum_{i=1}^{C}\sum_{j=1}^{h}L_{1}({Exp}_{i,j,:},T_{i,j,:})$$

$${Exp}_{i,j}=\sum_{k=1}^{w}k\cdot {Prob}_{i,j,k}$$

$${Prob}_{i,j,:}=softmax(P_{i,j,1:w})$$

where ${Prob}_{i,j,:}$ represents the probability distribution of the location of the jth lane line of the ith lane line, k represents the position of the cell in a row, and $L_{1}$ denotes $L_{1}$ loss.

**Figure 7:**
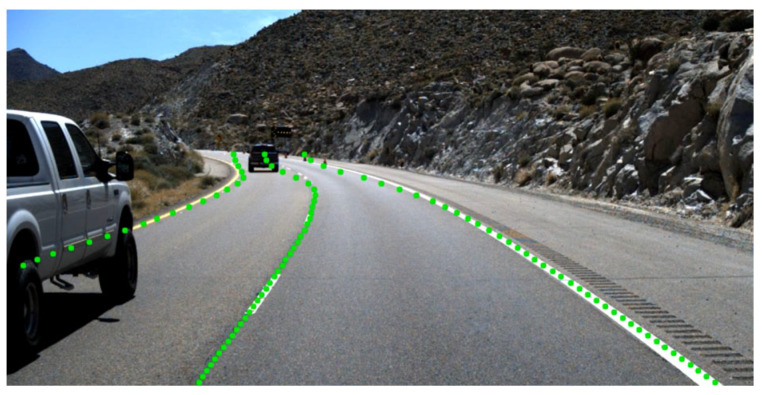
The structural loss in Ultra Fast Lane Detection causes the predicted point at the end of the lane line to be shifted to the right due to overfitting.

Structural loss: In the Ultra Fast method, the structural loss is

$$L_{shp}^{\prime }=\sum_{i=1}^{C}\sum_{j=1}^{h}{\left||P_{i,j}-P_{i,j+1}|\right|}_{1}$$

where $P_{i,j}$ represents the w+1 dimensional vector representing the lane line position predicted by the jth line of the ith lane line, and the magnitude of the change in the predicted positions of the upper and lower lane lines is obtained by calculating the difference between the predicted positions of the upper and lower lane lines of the same lane line and performing the $L_{1}$ normalization of the same lane line to yield the size of the change in the predicted positions of the upper and lower lane lines. It utilizes the a priori knowledge that lane lines are thin and continuous to design the structural loss function, aiming to make the predicted lane lines conform to the continuity, which can improve the model’s accuracy while speeding up the training speed, but there are still a lot of problems in the actual training, as shown in Figure 7, for example.

The lane lines captured by the front-view camera in real road conditions, as shown in Figure 3, Figure 4, Figure 5, Figure 6 and Figure 7, are usually inclined, which can cause the model to have a large value of the total loss function even though the model has achieved the desired training effect, leading to the overfitting problem in the model. And the row classification method predicts the lane line if there is no lane line in a row, and then the predicted position is the w+1st cell due to the existence of shape loss, which will cause the predicted point at the end of the lane line to be shifted to the right due to overfitting. In order to solve the overfitting problems caused by the original shape loss function, we improved the shape loss. Specifically for the overfitting problem, we want the distance between the top and bottom rows of the prediction result to be less than a threshold constant τ to indicate that the predicted lane lines are already continuous; in order to avoid the overfitting problem when the predicted lane lines are already continuous, the shape loss is no longer in effect. And in order to avoid the problem that the end of the lane line is shifted to the right due to overfitting by the shape loss, we add a new condition for the shape loss; when the prediction result is that the line does not contain lane lines in this row (the predicted position of the lane line is the w+1st cell), then the operation of the shape loss is not taken into account. The improved formula is as follows.

$$IF:P_{i,j},\;P_{i,j+1}=w+1\;\;\;\;{Lshp}_{i,j}=0$$

$$Else:{Lshp}_{i,j}=\left\{\begin{array}{cc} {\left||P_{i,j}-P_{i,j+1}|\right|}_{1} & P_{i,j}-P_{i,j+1}>\tau \\ 0 & P_{i,j}-P_{i,j+1}\leq \tau \end{array}\right.$$

$$L_{shp}=\sum_{i}^{C}\sum_{j}^{h}{Lshp}_{i,j}$$

where $P_{i,j}$ represents the w+1 dimensional vector representing the lane line position predicted by the jth line of the ith lane line, ${Lshp}_{i,j}$ represents the shape loss component computed by the jth line of the ith lane line, and $L_{shp}$ represents the total shape loss value.

For the auxiliary branch of the instance-based segmentation method, we use the cross-entropy function as its loss function $L_{seg}$.

The final total loss function is

$$L=aL_{cls}+bL_{exp}+cL_{shp}+dL_{seg}$$

where L is the total loss function, $L_{cls}$ is the categorization loss, $L_{exp}$ is the expectation loss, $L_{shp}$ is the shape loss, $L_{seg}$ is the auxiliary branching loss, and a,b,c,and d are the constants used as coefficients for each of the four losses.

## 4. Results

### 4.1. Datasets

For our experimental evaluations, we opted for the Tusimple dataset [29] and CULane dataset, which are widely acknowledged as the predominant datasets in the domain of lane detection.

**Figure 8:**
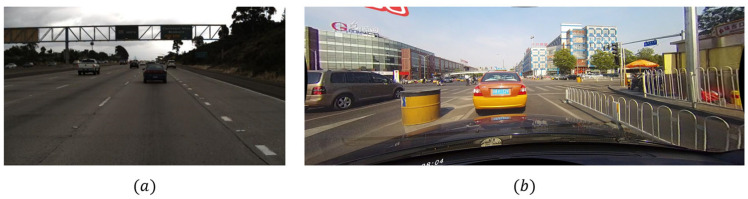
(**a**) shows a sample image from the Tusimple dataset, while (**b**) shows a sample image from the CULane dataset.

The Tusimple dataset contains 3626 training and 2782 testing video clips, each with 20 frames. The camera view aligns with the travel direction. Annotations, emphasizing lane marking folds, are in JSON format. The images are standardized at 1280 × 720 pixels. Figure 8 illustrates the dataset’s image data for context, as shown in Figure 8a.

The CULane dataset [30] is a widely used dataset for lane detection tasks, and the data are collected by the Multimedia Laboratory of the Chinese University of Hong Kong. It comprises a total of 98,000 images, encompassing eight challenging scenarios such as congestion, nighttime, no lane markings, and shadows. The data were collected using six taxis in Beijing, with a maximum of four lane lines marked per image. The image size is 1640 × 590 pixels, as shown in Figure 8b.

### 4.2. Evaluation Metrics

For the Tusimple dataset, we used the accuracy rate as an evaluation metric, which is formulated as

(1) $$accuracy=\frac{\sum_{clip}C_{clip}}{\sum_{clip}S_{clip}},$$

where $C_{clip}$ is the number of correctly predicted points, and $S_{clip}$ is the number of labeled value points.

The CULane dataset employs the End Point method to determine the accuracy of predictions by judging whether the distance between the endpoints of the lines and their enclosed area exceed a certain threshold.

### 4.3. Implementation Details

We chose ResNet-18 and ResNet-34 as the backbone network of our model, whose input receives images with a size of 288 × 800, so we needed to transform the input image to a size of 288 × 800 first to adapt to the network input during training or prediction. And we utilized a six-layer PoolFormer encoder along with a four-layer Transformer decoder. For the Tusimple dataset, it is necessary to preset four row anchors of 56 rows and 101 columns between 160 and 710 pixels of the image; our experiments set the object queries to a matrix of 56 × 512 in size, and the four FFNs were set to have an input dimension of 512 and an output dimension of 101. For the CULane dataset, it is necessary to preset four row anchors of 18 rows and 201 columns between 260 and 530 pixels; our experiments set the object queries to a matrix of 18 × 512 in size, and the four FFNs were set to have an input dimension of 512 and an output dimension of 201. During training, we used the Adam optimizer with a learning rate of 4 × 10^−4^ and trained the model using the cosine decay learning rate strategy. The Transformer encoder–decoder in the row classification branch consists of six encoders with four decoders. The loss function part $L=aL_{cls}+bL_{exp}+cL_{sim}+dL_{seg}$, a,b,c,d consists of values of 1,1,0.5,1. The method was built using the PyTorch framework, training was carried out on the Google Colaboratory platform, and Nvidia RTX 3080 GPU was used for the testing part.

### 4.4. Ablation Experiments

Ablation experiments were meticulously conducted on the Tusimple dataset to scrutinize the Transformer module, auxiliary branch, and loss function individually, thereby substantiating the efficacy of our proposed network. The outcomes of these ablation experiments are systematically presented in Table 1. The quantitative results for each module were derived from experiments conducted under consistent training parameters, exploring various combinations of modules. The analysis of Table 1 reveals noteworthy insights: in comparison to the Baseline (utilizing traditional segmentation methods), the novel network structure, incorporating the Transformer architecture, exhibits a substantial enhancement in performance. Furthermore, the incorporation of an auxiliary branch grounded in Feature-aligned Pyramid Network (FaPN) principles, coupled with a meticulously designed loss function, yields appreciable improvements in prediction accuracy. This comprehensive evaluation underscores the significance and effectiveness of the proposed network components in advancing the state of the art in lane detection tasks.

### 4.5. Contrast Experiment

The evaluation of our model on the Tusimple dataset and CULane dataset utilized ResNet-18 and ResNet-34 as distinct backbone networks. A comparative analysis was conducted against several existing methodologies, encompassing segmentation-based, row-anchor-based, line-anchor-based, curve-based, and key-point-based approaches. The outcomes of these comparisons, specifically pertaining to the Tusimple dataset, are systematically presented in Table 2. This examination serves to gauge the relative performance of our model in contrast to established methodologies across various lane detection paradigms, providing a comprehensive insight into its efficacy and positioning within the broader landscape of lane detection techniques (Table 3).

## 5. Conclusions

We present a novel lane detection methodology that amalgamates the row-anchor-based approach with a Transformer structure, yielding superior accuracy while concurrently ensuring real-time performance. Our method achieves a rapid prediction speed of 129 FPS (the single prediction time of the model on RTX3080 is 15.72 ms). While our method may not achieve the highest accuracy, it excels in meeting the real-time demands of lane detection in limited in-vehicle hardware. For example, the instance segmentation-based method shown in Table 2, despite its high accuracy, lacks the necessary real-time performance, which does not meet the needs of the displayed situation. Conversely, our approach balances both real-time performance and accuracy, making it more suitable for practical lane detection tasks.

Furthermore, our method demonstrates the viability of incorporating pooling as the transformer structure for the token model in the row-anchor-based lane detection method. This innovation enables future lane detection methods to leverage the transformer structure without concerns regarding compromised real-time performance stemming from an extensive parameter count.

Our proposed transformer encoder–decoder structure facilitates the extraction of global features, enhancing the delineation of lane features and effectively addressing challenges related to lane line occlusion. Additionally, the incorporation of a Feature-aligned Pyramid Network (FaPN)-based auxiliary branch contributes to improved detection accuracy without compromising the inference speed. Furthermore, our model introduces an enhanced loss function to rectify the structural deficiencies in row-anchor-based classification methods, thereby further elevating detection accuracy. Demonstrating superior performance on the Tusimple dataset, our model adeptly navigates the delicate balance between real-time processing and accuracy, affirming its efficacy in the realm of lane detection.

## Figures and Tables

**Table 1:** Ablation experiments.

Accuracy	Baseline	Transformer	Auxiliary Segmentation	New Loss
92.84%	√			
93.82		√		
94.11		√	√	
95.61%		√		√
96.16%		√	√	√

**Table 2:** Comparative study of Tusimple dataset.

Type	Method	Accuracy%	FPS	FP%	FN%	Device
Instance segmentation-based	SCNN (VGG-16)	96.53	7.5	6.17	1.80	GTX Titan Black
	FRLD (0.970)	96.30	49	-	-	-
	ROLD [31]	96.70	77	3.59	2.82	-
	SSLD [32]	97.08	110	3.90	1.80	GTX1080Ti.
Row-anchor-based	UFLD (ResNet-18)	95.82	323	19.05	3.92	GTX1080Ti.
	UFLDv2 (ResNet-18)	95.65	330	3.06	4.61	RTX 3090
	CondLaneNet (ResNet-18)	95.84	220	2.18	3.80	RTX 2080
Line-anchor-based	CLRNet (ResNet-18)	96.84	119	2.28	1.92	GTX 1080Ti
Curve-based	BézierLaneNet (ResNet-18)	95.41	210	5.30	4.60	RTX 2080Ti
Key-point-based	GANet-S	95.96	153	1.97	2.62	Tesla-V100
Our	Our (ResNet-18)	96.01	129	17.1	3.51	RTX 3080
	Our (ResNet-32)	96.16	118	18.3	3.62	RTX 3080

**Table 3:** Comparative study of CULane dataset.

Method	Total	Normal	Crowd	Dazzle	Shadow	No Line	Arrow	Curve	Night
SCNN (VGG-16)	71.60	90.60	69.70	58.50	66.90	43.40	84.10	64.40	66.10
UFLD (ResNet-18)	68.40	87.70	66.00	58.40	62.80	40.20	81.00	57.90	62.10
UFLDv2 (ResNet-18)	72.30	90.70	70.20	59.50	69.30	44.20	85.70	69.50	66.70
CondNet (ResNet-18)	78.14	92.87	75.79	70.72	80.01	52.39	89.37	72.40	73.23
CLRNet (ResNet-18)	79.58	93.30	78.33	73.71	79.66	53.14	90.25	71.56	75.11
BézierNet (ResNet-18)	73.67	90.22	71.55	62.49	70.91	45.30	84.09	58.98	68.70
GANet-S	78.79	93.24	77.16	71.24	77.88	53.59	89.62	75.92	72.75
O2SFormer (ReNet-18) [33]	76.07	91.89	73.86	70.40	74.84	49.83	86.08	68.68	70.74
SSLD [32]	73.20	90.80	72.30	-	-	-	-	67.2	-
Our (ResNet-18)	72.25	91.47	69.20	59.25	67.77	44.21	84.72	69.26	66.58
Our (ResNet-32)	73.02	92.72	70.67	60.29	69.89	45.93	86.69	72.34	68.27

## Data Availability

Data are contained within the article.
